# Effect of a Digital-Driven Physician-Pharmacist Collaborative Model for Diabetes in Primary Health Care: Cluster Randomized Trial

**DOI:** 10.2196/77470

**Published:** 2026-03-13

**Authors:** Jie Xiao, Qing Wang, Shenglan Tan, Lei Chen, Daxiong Xiang, Haiyan Yuan, Xia Li, Shuting Huang, Bingjie Tang, Yan Guo, Haiying Huang, Danhui Zhao, Yue Li, Li Wang, Qun Li, Juan Liu, Ping Xu

**Affiliations:** 1 Department of Pharmacy Second Xiangya Hospital Central South University Changsha China; 2 Institute of Clinical Pharmacy Second Xiangya Hospital Central South University Changsha China; 3 Department of Endocrine Second Xiangya Hospital Central South University Changsha China; 4 Department of Pharmacy Taoyuan People's Hospital Changde China; 5 Department of Pharmacy The People's Hospital of Liuyang Changsha China; 6 Department of Pharmacy The First People's Hospital of Pingjiang Yueyang China; 7 Department of Pharmacy People's Hospital of Ningxiang Changsha China; 8 Department of Pharmacy Yueyang Central Hospital Yueyang China; 9 Department of Pharmacy The Second People's Hospital of Huaihua Huaihua China; 10 Department of Pharmacy Yiyang Central Hospital Yiyang China

**Keywords:** diabetes mellitus, physician-pharmacist collaboration, central obesity, glycemic control, atherosclerotic cardiovascular risk, cost-effectiveness analysis

## Abstract

**Background:**

Evidence-based physician-pharmacist collaborative clinics have demonstrated significant short-term benefits for patients with type 2 diabetes (T2D), but their long-term effectiveness remains unclear, especially in primary health care settings.

**Objective:**

This study aimed to explore the long-term effectiveness and cost-effectiveness of a novel, digital-driven, multifaceted physician-pharmacist collaborative model for managing patients with T2D in underresourced settings.

**Methods:**

We conducted a 12-month cluster randomized controlled trial from May 2021 to December 2022 across 6 primary health care settings in China. Guided by the theory of planned behavior, the intervention involved routine therapy from physicians along with pharmaceutical interventions from pharmacists. These were delivered through a combination of face-to-face visits and mobile health care. The intervention group received 4 face-to-face visits and biweekly remote education sessions over the 12 months. We conducted intention-to-treat analyses to estimate differences in clinical and behavior indicators between the intervention and control groups. Primary outcomes included glycosylated hemoglobin and 10-year atherosclerotic cardiovascular risk. Data were analyzed using adjusted generalized estimation equations.

**Results:**

This study included 574 patients (291 in the intervention group and 283 in the control group). Over 12 months, patients in the intervention group had significant reductions in hemoglobin A_1c_ (–2.57 vs –1.96, respectively; *P*<.001; 95% CI –1.027 to –0.238) and 10-year atherosclerotic cardiovascular risk (–1.35 vs 0.01, respectively; *P*<.001; 95% CI –1.690 to –0.630) compared with the control group. Substantial improvements were also observed in several secondary outcomes, including fasting blood glucose, 2-hour postprandial blood glucose, waist circumference, waist-to-hip ratio, blood pressure, triglyceride, and total cholesterol. Total diabetes-related costs decreased, and patient satisfaction improved significantly in the intervention group. There were no significant differences in BMI, high-density lipoprotein, or low-density lipoprotein.

**Conclusions:**

These findings suggest that the physician-pharmacist collaborative model could improve the long-term quality and efficiency of T2D management and reduce medical costs in underresourced areas globally. Patients with T2D, especially those with central obesity or high cardiovascular risk, may benefit more from collaborative clinics.

**Trial Registration:**

Chinese Clinical Trial Registry ChiCTR2000031839; https://www.chictr.org.cn/showproj.html?proj=51910

## Introduction

The global epidemic of diabetes, particularly type 2 diabetes (T2D), has caused severe public health challenges worldwide [1]. The International Diabetes Federation estimates that the prevalence of diabetes worldwide was 10.5% in 2021 and would increase to 12.2% by 2045 due to rapid lifestyle changes and aging of the population [2]. China has the largest population of people living with diabetes worldwide, with an estimated 1.39 million deaths attributable to diabetes or its complications in 2021, and this number continues to rise rapidly [2]. As in other countries, most of the population with diabetes in China has T2D, and half of them present with complications, leading to a huge economic burden [3]. This burden is exacerbated in resource-limited settings, where health care systems are often underequipped to manage the long-term complexities of the disease [1].

Effective, long-term management of T2D is therefore crucial to mitigating these complications and associated costs [4]. Diabetes management is multidimensional, including a low-energy diet, physical activity, medication, diabetes education, and continuous glucose monitoring [4-6]. In vast rural areas of China, primary health care centers are the preferred institutions for more than 900 million people, where diabetes management is still dominated by physicians [7]. However, diabetes management in rural areas in China is currently insufficient due to limited resources and staff shortage in primary health care centers [8]. Moreover, patients in rural areas generally have lower educational background and socioeconomic status, which necessitates more detailed treatment and management support, which puts further strain on the capacity of the current physician-led care model [9].

To address these issues, innovative team-based care models have shown promise [10-13]. One well-established model is the physician-pharmacist collaboration, which improves the quality of outpatient services and reduces physicians’ workload by involving pharmacists in medication counseling and patient education [14,15]. However, due to the limited reach and accessibility of in-person pharmaceutical services, it could be difficult to implement established pharmaceutical intervention programs in underresourced areas [16]. Mobile health (mHealth) interventions offer a promising way to enhance and sustain collaborative care in these contexts [17]. Evidence shows that mHealth tools, including medication reminders, mobile apps for tracking, and remote monitoring, can improve medication adherence, self-management behaviors, and clinical outcomes in diabetes care, thereby alleviating the workload of health care providers [18,19]. An mHealth-based physician-pharmacist collaboration model could be adopted in underresourced settings.

Previous studies have demonstrated that the intervention programs based on the theory of planned behavior (TPB) contribute to change behavior patterns, but few studies have tailored the method to comprehensive intervention for diabetes management in underresourced settings [20,21]. Therefore, we have developed a digital-driven, multifaceted intervention program based on physician-pharmacist collaborative models, combining face-to-face visits and mHealth support. We conducted a cluster randomized controlled trial to evaluate the long-term effectiveness of the intervention on hemoglobin A_1c_ (HbA_1c_), 10-year atherosclerotic cardiovascular disease (ASCVD) risk, body weight, blood pressure, lipids, and cost-effectiveness of the physician-pharmacist collaborative model for T2D management in primary health care centers in China.

## Methods

### Study Design

The cluster randomized controlled trial was conducted from May 2021 to December 2022 in 6 health care centers in Hunan province, China. A trial protocol was reported in a previous study [22]. Hunan province is located in the center of China, with middle-level economy and general medical condition. To ensure representativeness, all 88 Hunan counties were divided into 3 subgroups by per capita income level, namely, high, medium, and low incomes. We then sent invitations to 27 primary health care centers (the largest by outpatient volume in their respective counties) across these strata. Of the 18 centers that agreed to participate, we randomly selected 2 centers from each income stratum. Within each stratum, the 2 selected centers were then randomly allocated to the intervention and control groups (1 per group), resulting in 3 centers in the intervention group and 3 in the control group. All eligible patients attending these centers received care according to their center’s allocation. This study was reported following the CONSORT (Consolidated Standards of Reporting Trials) extension for cluster trials (Multimedia Appendix 1). We held progress meetings monthly and used fidelity checklists to monitor implementation (Multimedia Appendix 2).

### Randomization and Masking

Randomization was performed at the level of the health care center (cluster). Using a standard randomization protocol with a computer-based random sequence generation, an independent statistician performed the randomization [23]. This involved randomly selecting the 6 study centers from the 18 consenting centers, followed by randomly allocating the selected centers to the intervention and control groups with balanced allocation. Because the intervention required knowledge of patient allocation, blinding of participants was not feasible; however, data processors were blinded to group assignment.

### Participants

The study sample included patients with T2D who were aged ≥18 years, had HbA_1c_ levels >7.5% in the past 2 months, and provided contact information. Patients were excluded if they were pregnant or had end-stage renal failure, dementia, psychiatric disorders, cancer, congestive heart failure, or pancreatitis. All eligible patients were required to provide written informed consent before participation in the study.

### Intervention and Procedures

#### Pharmacist Training and Qualification

Pharmacists participating in the study had worked as clinical pharmacists for >2 years, held standardized training qualifications, and completed a 3-month comprehensive training course at the Second Xiangya Hospital. The training courses mainly cover 2 aspects: diabetes management and cooperative skills. Topics included knowledge of diabetes, hypoglycemic agents, treatment target, interpretation of laboratory tests, medication adherence, diet, exercise, and lifestyle. Detailed training content is available in Table S1 in Multimedia Appendix 3 [24]. Intermediate and final tests are used to assess learning, and those who fail the tests were excluded from the study [22].

#### Intervention Group

Centers in the intervention group provided routine physician-led therapy combined with pharmacist-delivered pharmaceutical care through outpatient visits at baseline and at the third, sixth, ninth, and 12th months, along with biweekly remote education via WeChat (the most popular social media platform in China [25,26]) throughout the 12-month study period. The pharmaceutical intervention program was based on the TPB framework and included 3 areas: attitude toward the behavior, subjective norm, and perceived behavior control (Textbox 1). Well-trained pharmacists provided multifaceted pharmaceutical care at each follow-up visit, which lasted at least 20 minutes; handed out health education manuals at the end of visits; and conducted biweekly remote health education through WeChat to regulate the patient attitude toward treatment. Multifaceted pharmaceutical care included diabetes education, medication guidance, lifestyle intervention, treatment of adverse drug reactions, and complication identification, while remote health education involved pharmaceutical consultation, structured educational messages, and regular medication reminders (Table S2 in Multimedia Appendix 3). Subjective norm was achieved through peer support and family support to strengthen patient behavior intention. Meanwhile, pharmacists set health goals for patients and strengthened their medication knowledge to improve the perception of treatment effects.

**The intervention program based on the theory of planned behavior.**

**Attitude toward the behavior**

- Face-to-face pharmaceutical care at each visit, including diabetes education, medication guidance, lifestyle intervention, adverse drug reaction management, and complication identification
- Health education manuals were distributed, covering diabetes knowledge, hazards of complications, introduction of commonly used drugs, healthy lifestyle, and other related knowledge
- Health education was delivered through the WeChat group, including answering questions from patients and their families; providing guidance on daily management; offering disease education; and sharing pharmaceutical knowledge, dietary advice, and exercise plans

**Subjective norm**

- Peer support, which allows patients to interact with each other within their peer group in WeChat
- Family support, including diabetes care, rational drug use, healthy lifestyle, and other related knowledge taught by pharmacists

**Perceived behavior control**

- Formulation of health goals, including short-term and long-term health goals
- Medication consultation services, including dose adjustment, medication duration, drug interactions, and adverse drug reactions

#### Control Group

Patients in the control centers received usual treatment and management by physicians, with no pharmacist available to provide additional patient education. After the study, all patients received a diabetes education manual.

### Outcomes

The primary outcomes were HbA_1c_ and 10-year ASCVD risk over 12 months, while glycemic measurements, weight-related measurements, blood pressure, lipid indicators, patient satisfaction, and costs were secondary outcomes. HbA_1c_ provides a better reflection of the patient’s average blood glucose level over a 3-month period. The 10-year ASCVD risks were calculated by the Prediction for atherosclerotic cardiovascular disease risk in China model [27]. The biomarkers of glycemic measurements include fasting blood glucose (FBG) and 2-hour postprandial blood glucose (PBG2h). Weight-related measurements included BMI, waist circumference (WC), and waist-to-hip ratio (WHR). The indicators of blood pressure included systolic blood pressure and diastolic blood pressure. The biomarkers of blood lipid status include triglyceride, total cholesterol (TC), high-density lipoprotein cholesterol, and low-density lipoprotein cholesterol (LDL-C). Patient satisfaction was assessed by the Diabetes Treatment Satisfaction Questionnaire, with a Cronbach alpha index of 0.717. The total cost of a patient’s treatment included the costs of drugs, registration, examinations, hospitalization, lost wages, and transportation related to diabetes care.

### Sample Size

On the basis of previous studies, we calculated that at 90% power and 5% significance level, the minimum detectable difference in HbA_1c_ in the intervention group was 0.76% with an SD of 1.0%, while minimum detectable difference in 10-year ASCVD risk was 3.98 [28,29]. The intraclass correlation coefficients at the center level were 0.03 for HbA_1c_ and 0.05 for 10-year ASCVD risk [30,31]. Considering the potential effects of clustering and loss to follow-up over 12 months, the study required a sample size of at least 288 patients in this study (144 in each group).

### Statistical Analysis

All analyses were conducted using a modified intention-to-treat approach, which excluded 26 patients who withdrew after randomization but before trial implementation. Measurement data were presented as means with SDs or median with IQRs, while enumeration data were presented as counts with percentages. Descriptive statistics were used to summarize the baseline demographic characteristics, while independent sample 2-tailed *t* tests and Mann-Whitney *U* tests were used to compare the differences between groups at baseline. Differences in measurements and costs over 12 months between the groups were detected by generalized estimating equation (GEE) analysis. GEE provides stable estimates of time, group, and interaction effects for repeated measurement data, while GEE model gave valid estimation under the assumption of missing at random. GEE models were adjusted for center, gender, age, comorbidity, smoking history, alcohol history, family history, education, income, occupation, and residence. The standard rate of control was referred to the comprehensive control target of T2D in China (Table S3 in Multimedia Appendix 3). Sensitivity analysis was performed using the per-protocol population, which included patients who adhered to the protocol and were not lost to follow-up. Missing data were minimal (<10% for primary outcomes) and were handled using GEE, which provides valid estimates under the missing-at-random assumption by using all available data without imputation. Additionally, 2-sided *P* values <.05 were considered significant for all analyses. SPSS Statistics (version 25.0; IBM Corp) and SAS (version 9.4; SAS Institute) were used.

### Ethical Considerations

This study was approved by the Clinical Research Ethics Committee of the Second Xiangya Hospital of Central South University (2019-213). All participants were informed of the study's purpose, procedures, potential risks, and benefits, and they provided written informed consent prior to enrollment. Strict measures were taken to protect participant privacy and confidentiality; all data were anonymized before analysis. As compensation for their participation, all enrolled patients received free laboratory examinations related to the study.

## Results

### Baseline Characteristics

**Figure 1:**
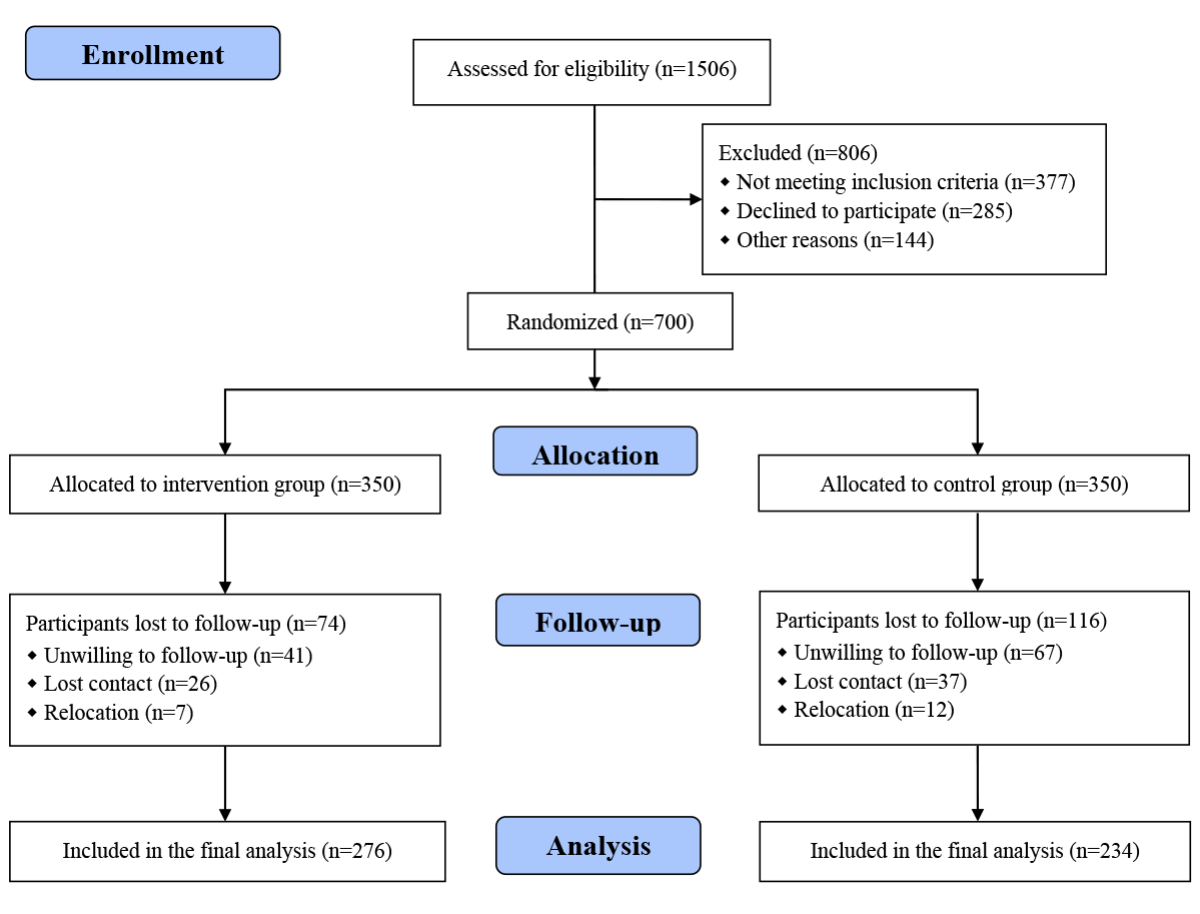
Flowchart of the trial.

The study was conducted in 6 centers (Table S4 in Multimedia Appendix 3) and included 574 patients, of whom 50.7% (n=291) participated in physician-pharmacist collaborative clinics (Table 1). The mean age of the enrolled patients was 55.0 (48.0 to 64.0) years and 52.4% (n=301) were male. Overall, 34% (n=195) of the patients had a history of smoking and 27.2% (n=156) a history of alcohol use. Baseline characteristics of patients were generally comparable between the 2 groups. Patient enrollment is shown in Figure 1.

**Table 1:** Patients’ demographic characteristics (N=574).

Characteristics				Overall		Intervention (n=291)		Control (n=283)
Age (years), median (IQR)				55.0 (48.0-64.0)		54.0 (49.0-60.0)		55.0 (46.0-67.0)
**Sex, n (%)**								
	Male			301 (52.4)		139 (47.8)		162 (57.2)
	Female			273 (47.6)		152 (52.2)		121 (42.8)
**Comorbidity, n (%)**								
	**Hypertension**							
		Yes	90 (15.7)		50 (17.2)		40 (14.1)	
	**Hyperlipidemia**							
		Yes	26 (4.5)		10 (3.4)		16 (5.7)	
	**Coronary heart disease**							
		Yes	20 (3.5)		11 (3.8)		9 (3.2)	
**History of smoking, n (%)**								
	Yes			195 (34)		84 (28.9)		111 (39.2)
**History of alcohol intake, n (%)**								
	Yes			156 (27.2)		83 (28.5)		73 (25.8)
**Family history, n (%)**								
	**Diabetes**							
		Yes	164 (28.6)		89 (30.6)		75 (26.5)	
	**Hypertension**							
		Yes	94 (16.4)		54 (18.6)		40 (14.1)	
	**Hyperlipidemia**							
		Yes	45 (7.8)		23 (7.9)		22 (7.7)	
**Education, n (%)**								
	Junior high school and below			318 (55.4)		168 (57.7)		150 (53)
	Senior high school			152 (26.5)		78 (26.8)		74 (26.1)
	College and above			104 (18.1)		45 (15.5)		59 (20.9)
**Income per month (CNY)^a^, n (%)**								
	<1000			96 (16.7)		54 (18.5)		42 (14.8)
	1000-3000			191 (33.3)		98 (33.7)		93 (32.9)
	>3000			287 (50)		139 (47.8)		148 (52.3)
**Occupation, n (%)**								
	Full-time job			395 (68.8)		215 (73.9)		180 (65.9)
	Part-time job or no job			179 (31.2)		76 (26.1)		103 (34.1)
**Residence, n (%)**								
	Rural area			321 (55.9)		151 (51.9)		170 (62.3)
	Town or urban area			253 (44.1)		140 (48.1)		113 (37.7)
HbA_1c_^b^, mean (SD)				9.83 (2.02)		9.65 (1.87)		10.02 (2.15)
10-year ASCVD^c^ risk, mean (SD)				7.56 (5.87)		7.05 (4.92)		8.06 (6.65)
FBG^d^ (mmol/L), mean (SD)				10.65 (4.16)		10.60 (3.60)		10.69 (4.66)
PBG2h^e^ (mmol/L), mean (SD)				15.19 (5.27)		15.34 (4.64)		15.03 (5.84)
BMI (kg/m^2^), mean (SD)				24.87 (3.33)		24.73 (3.38)		25.02 (3.28)
**WC^f^ (cm), mean (SD)**								
	Male			91.92 (9.34)		91.17 (8.89)		92.60 (9.69)
	Female			88.73 (10.04)		88.62 (10.23)		88.88 (9.86)
**WHR^g^, mean (SD)**								
	Male			0.927 (0.062)		0.924 (0.061)		0.930 (0.062)
	Female			0.927 (0.078)		0.927 (0.076)		0.927 (0.078)
SBP^h^ (mm Hg), mean (SD)				133.38 (18.33)		132.53 (16.05)		134.26 (20.41)
DBP^i^ (mm Hg), mean (SD)				81.20 (9.90)		80.47 (9.03)		81.96 (10.71)
Triglyceride (mmol/L), mean (SD)				2.80 (3.15)		3.09 (3.87)		2.53 (2.20)
TC^j^ (mmol/L), mean (SD)				4.98 (1.56)		5.12 (1.74)		4.84 (1.36)
HDL-C^k^ (mmol/L), mean (SD)				1.30 (0.57)		1.30 (0.53)		1.30 (0.60)
LDL-C^l^ (mmol/L), mean (SD)				2.94 (1.11)		2.96 (1.25)		2.92 (0.94)

^a^CNY 1=US $0.145.

^b^HbA_1c_: hemoglobin A_1c_.

^c^ASCVD: atherosclerotic cardiovascular disease.

^d^FBG: fasting blood glucose.

^e^PBG2h: 2-hour postprandial blood glucose.

^f^WC: waist circumference.

^g^WHR: waist-to-hip ratio.

^h^SBP: systolic blood pressure.

^i^DBP: diastolic blood pressure.

^j^TC: total cholesterol.

^k^HDL-C: high-density lipoprotein cholesterol.

^l^LDL-C: low-density lipoprotein cholesterol.

### Primary Outcomes

At 12 months, the reduction in HbA_1c_ was significantly greater in the intervention group (−2.57%) than in the control group (−1.96%; β=−0.632; *P*<.001; 95% CI −1.027 to −0.238). Detailed information is shown in Table 2. Correspondingly, the HbA_1c_ control rates increased markedly from baseline to 12 months in both groups, but the improvement was significantly greater in the intervention group (from 0% to 53.61%) than in the control group (from 1.77% to 38.52%; *P*=.001). Refer to Table S5 in Multimedia Appendix 3 for full details. The 10-year ASCVD risk decreased significantly in the intervention group compared with the control group over the study period (β=−1.206; *P*<.001; 95% CI −1.730 to −0.68).

**Table 2:** The 12-month effectiveness of the physician-pharmacist collaborative clinics on clinical outcomes.

Variables			Intervention group, mean (SD)		Control group, mean (SD)		Unadjusted effect						Adjusted effect^a^				
							*P* value^b^			β (95% CI)			*P* value			β (95% CI)	
**HbA_1c_^c^**								<.001			−0.632 (−1.026 to −0.237)			<.001			−0.632 (−1.027 to −0.238)
	Baseline	9.65 (1.87)		10.02 (2.15)													
	3 months	7.93 (1.53)		8.43 (2.19)													
	6 months	7.76 (1.63)		8.15 (1.86)													
	9 months	7.55 (1.48)		8.18 (1.87)													
	12 months	7.08 (1.13)		8.06 (2.02)													
**10-year ASCVD^d^ risk**								<.001			−1.160 (−1.690 to −0.630)			<.001			−1.206 (−1.730 to −0.682)
	Baseline	7.05 (4.92)		8.06 (6.65)													
	12 months	5.70 (4.05)		8.07 (6.37)													
**FBG^e^**								.29			−0.592 (−1.363 to 0.178)			.27			−0.602 (−1.372 to 0.169)
	Baseline	10.60 (3.60)		10.69 (4.66)													
	3 months	8.38 (2.54)		9.03 (3.32)													
	6 months	8.40 (2.77)		8.92 (2.67)													
	9 months	8.02 (2.43)		8.31 (2.28)													
	12 months	7.58 (2.09)		8.22 (2.67)													
**PBG2h^f^**								.03			−1.234 (−2.338 to −0.129)			.03			−1.210 (−2.315 to −0.106)
	Baseline	15.34 (4.64)		15.03 (5.84)													
	3 months	12.38 (3.89)		12.03 (4.54)													
	6 months	11.60 (3.67)		11.45 (4.63)													
	9 months	11.36 (3.42)		11.06 (4.07)													
	12 months	10.44 (3.66)		11.17 (4.10)													
**BMI**								.21			−0.238 (−0.528 to 0.051)			.23			−0.236 (−0.527 to 0.054)
	Baseline	24.73 (3.38)		25.02 (3.28)													
	3 months	24.49 (3.22)		24.68 (3.38)													
	6 months	24.37 (3.13)		24.89 (3.16)													
	9 months	24.23 (3.12)		24.73 (3.29)													
	12 months	24.28 (3.14)		24.77 (3.36)													
**WC^g^**								<.001			−3.570 (−4.200 to −2.940)			<.001			−3.557 (−4.185 to −2.929)
	Baseline	89.83 (9.68)		90.98 (9.89)													
	3 months	89.06 (9.67)		91.46 (9.83)													
	6 months	87.09 (9.21)		91.82 (9.46)													
	9 months	87.02 (9.42)		92.50 (9.13)													
	12 months	86.84 (9.24)		91.92 (9.85)													
**WHR^h^**								<.001			−0.020 (−0.025 to −0.015)			<.001			−0.020 (−0.024 to −0.013)
	Baseline	0.926 (0.069)		0.929 (0.070)													
	3 months	0.920 (0.073)		0.930 (0.080)													
	6 months	0.911 (0.068)		0.932 (0.073)													
	9 months	0.909 (0.070)		0.938 (0.073)													
	12 months	0.910 (0.068)		0.933 (0.067)													
**SBP^i^**								.007			−3.614 (−6.239 to −0.989)			.008			−3.556 (−6.187 to −0.925)
	Baseline	132.53 (16.05)		134.26 (20.41)													
	3 months	130.21 (13.26)		132.67 (16.37)													
	6 months	128.53 (12.14)		132.92 (14.10)													
	9 months	127.28 (11.24)		134.30 (17.08)													
	12 months	127.30 (11.70)		132.54 (15.20)													
**DBP^j^**								.01			−2.683 (−4.342 to −1.024)			.01			−2.639 (−4.295 to −0.983)
	Baseline	80.47 (9.03)		81.96 (10.71)													
	3 months	78.72 (9.04)		81.77 (10.64)													
	6 months	77.51 (8.16)		81.53 (9.45)													
	9 months	77.12 (8.99)		82.09 (9.94)													
	12 months	77.06 (7.94)		81.26 (8.52)													
**Triglyceride**								.01			−0.815 (−1.293 to −0.337)			.01			−0.814 (−1.293 to −0.335)
	Baseline	3.09 (3.87)		2.53 (2.20)													
	3 months	2.52 (2.54)		2.40 (2.37)													
	6 months	2.37 (2.24)		2.44 (2.70)													
	9 months	2.19 (2.30)		2.20 (1.53)													
	12 months	1.93 (1.40)		2.24 (1.53)													
**TC^k^**								.001			−0.544 (−0.804 to 0.284)			.001			−0.540 (−0.798 to −0.282)
	Baseline	5.12 (1.74)		4.84 (1.36)													
	3 months	4.71 (1.18)		4.60 (1.09)													
	6 months	4.43 (0.96)		4.47 (1.14)													
	9 months	4.16 (0.77)		4.28 (1.12)													
	12 months	4.12 (0.86)		4.37 (0.96)													
**HDL-C^l^**								.24			0.109 (0.014 to 0.205)			.28			0.109 (0.013 to 0.204)
	Baseline	1.30 (0.53)		1.30 (0.60)													
	3 months	1.33 (0.66)		1.26 (0.37)													
	6 months	1.32 (0.45)		1.24 (0.49)													
	9 months	1.33 (0.49)		1.25 (0.49)													
	12 months	1.36 (0.39)		1.25 (0.56)													
**LDL-C^m^**								.40			−0.043 (−0.228 to 0.143)			.42			−0.048 (−0.232 to 0.137)
	Baseline	2.96 (1.25)		2.92 (0.94)													
	3 months	2.74 (0.96)		2.85 (0.93)													
	6 months	2.78 (0.95)		2.78 (0.81)													
	9 months	2.79 (0.93)		2.77 (0.77)													
	12 months	2.78 (0.83)		2.78 (0.80)													
**Satisfaction**								<.001			−6.271 (−7.191 to −5.352)			<.001			−4.314 (−5.176 to −3.451)
	12 months	32.43 (4.38)		26.16 (5.59)													

^a^Adjusted for center, gender, age, comorbidity, history of smoking and alcohol, family history, education, income, occupation, and residence.

^b^Estimated group-by-time interaction effects from generalized estimation equation; time effect and group effect are displayed in Table S6 in Multimedia Appendix 3.

^c^HbA_1c_: hemoglobin A_1c_.

^d^ASCVD: atherosclerotic cardiovascular disease.

^e^FBG: fasting blood glucose.

^f^PBG2h: 2-hour postprandial blood glucose.

^g^WC: waist circumference.

^h^WHR: waist-to-hip ratio.

^i^SBP: systolic blood pressure.

^j^DBP: diastolic blood pressure.

^k^TC: total cholesterol.

^l^HDL-C: high-density lipoprotein cholesterol.

^m^LDL-C: low-density lipoprotein cholesterol.

### Secondary Outcomes

FBG levels decreased significantly more in the intervention group (mean 7.58, SD 2.09 mmol/L) than in the control group (mean 8.22, SD 2.67 mmol/L; *P*<.001), although the group-by-time interaction effect was not significant (β=−0.602; *P*=.27; 95% CI −1.372 to 0.169). Several other secondary outcomes showed significant group-by-time interaction effects favoring the intervention group, including PBG2h (β=−1.210; *P*=.03; 95% CI −2.315 to −0.106), systolic blood pressure (β=−3.556; *P*=.008; 95% CI −6.187 to −0.925), diastolic blood pressure (β=−2.639; *P*=.01; 95% CI −4.295 to −0.983), triglyceride (β=−0.814; *P*=.01; 95% CI −1.293 to −0.335), and TC (β=−0.540; *P*=.001; 95% CI −0.798 to −0.282). However, there were no significant differences in high-density lipoprotein cholesterol (β=0.109; *P*=.28; 95% CI 0.013-0.204) and LDL-C (β=−0.048; *P*=.42; 95% CI −0.232 to 0.137) between the 2 groups in the long-term interventions. Detailed data are presented in Table S6 in Multimedia Appendix 3.

Significant improvements in indicators of central obesity were also observed. WC (β=−3.557; *P*<.001; 95% CI −4.185 to −2.929) and WHR (β=−0.020; *P*<.001; 95% CI −0.024 to −0.013) decreased significantly in the intervention group, showing a significant effect on weight loss in obese people. However, the change in BMI (β=−0.236; *P*=.23; 95% CI −0.527 to 0.054) was not significant between groups. Patients in the intervention group had a mean satisfaction score of 32.43 (SD 4.38), significantly higher than those in the control group with a mean score of 26.16 (SD 5.59; β=−4.314; *P*<.001; 95% CI −5.176 to −3.451).

### Economic Evaluation

Over the 12 months, the cost of diabetes care was significantly lower in the intervention group (mean 5505.86, SD 3939.86 CNY) than in the control group (mean 7902.61, SD 11,218.51 CNY; *P*=.01; Table 3). The costs of registration, examination, lost wages, and transportation of patients were significantly higher in the intervention group than in the control group, while the hospitalization costs were significantly lower, indicating a shift from inpatient care toward more routine outpatient management.

**Table 3:** The 12-month total costs (in CNY^a^) of patients in the 2 groups (N=574).

Variables	Overall, mean (SD)	Intervention (n=291), mean (SD)	Control (n=283), mean (SD)	*P* value
Drug cost	3986.64 (2268.41)	3835.58 (2006.62)	4141.97 (2503.41)	.12
Registration cost	47.40 (31.40)	56.01 (29.01)	38.54 (31.34)	<.001
Examination cost	520.67 (741.48)	550.88 (323.56)	490.14 (999.86)	<.001
Hospitalization cost	1935.12 (7983.22)	849.72 (3299.63)	3051.21 (10,762.68)	.01
Lost wages	160.87 (276.69)	167.56 (301.06)	154.00 (249.52)	.02
Transportation cost	41.85 (63.14)	56.54 (69.86)	26.75 (51.28)	<.001
Total cost	6687.53 (8440.00)	5505.86 (3939.86)	7902.61 (11,218.51)	.01

^a^CNY 1=US $0.145.

### Sensitivity Analysis

The per-protocol population included 510 patients who adhered to the protocol and were not lost to follow-up. There were no significant differences in demographic characteristics between the modified intention-to-treat and per-protocol populations (Table S7 in Multimedia Appendix 3). Notably, significant improvements in primary and secondary outcomes were detected in per-protocol population, including HbA_1c_, 10-year ASCVD risk, glycemic measurements, weight-related outcomes, blood pressure, and blood lipid parameters (*P*<.05; Tables S8-S10 in Multimedia Appendix 3).

## Discussion

### Principal Findings

This study reported a digital-driven, multifaceted physician-pharmacist collaborative model for diabetes treatment and management, adapted for underresourced settings. The study also evaluated the long-term effectiveness and cost-effectiveness of implementing physician-pharmacist collaborative models on participants with diabetes in primary health care. The intervention was found to significantly improve long-term clinical efficacy and associated costs for patients over 12 months in primary health care settings, with the positive effects becoming more significant over time. In all cases, the magnitude of the improvements implied benefits for patients and public health.

The intervention program, based on the TPB framework, aims to enhance self-management in patients with diabetes by facilitating the establishment of behavioral intentions and motivation, was well as stimulating positive therapeutic behaviors [21,32]. Previous studies have found that TPB serves to predict individuals’ motivation to engage in health behaviors, including exercise, health checks, disease screening, and health management behaviors [33]. However, most of the available studies are derived from high-income countries and regions, leaving very limited research on TPB-based interventions in rural populations with low education and economic status [21,33,34]. Our study suggests that the TPB-based intervention program contributes to the improvement of health behaviors among patients with diabetes in resource-limited areas, providing a strong basis for the widespread use of TPB in primary health care settings.

Our findings indicated a long-term advantage of physician-pharmacist collaborative models in improving HbA_1c_ and glycemic outcomes. The HbA_1c_ control rate in the intervention group increased to 51.45%, remarkably higher than the average control rate in rural China of 44.1% and close to the urban rate of 54.1%, suggesting a narrowing of the rural-urban disparities in diabetes treatment and management [35]. Moreover, there was an improvement in FBG at the early intervention, whereas the improvement in PBG2h was not observed until 12 months. This is supported by previous studies, showing that postprandial glycemic fluctuations tend to occur in patients with well-controlled diabetes, whereas patients with worsening diabetes have higher FBG [36-38]. Therefore, physician-pharmacist collaborative clinics have shown excellent management of glycemic outcomes in the early stages of diabetes, contributing to the delay of disease progression.

The results were consistent with other studies in high-income countries and regions, which have reported comprehensive improvements in blood glucose, blood pressure, and lipids in patients with diabetes [39,40]. Pharmacist-delivered services improved the quality of health care and reduced the burden of care, generating an annual return on investment of US $1.2 million to US $2.9 million [39]. A study of 2480 patients demonstrated that the pharmacist-physician collaborative care model resulted in significant improvements in HbA_1c_, blood pressure, and lipid levels [40]. However, it is controversial for improving cardiovascular outcomes, while long-term cardiovascular risk was unclear [15,41]. Few retrospective multicenter cohort studies showed that the collaborative care model effectively decreased the HbA_1c_ levels but did not improve cardiovascular risk in primary care settings [42,43]. The potential explanation is the absence of an explicit theoretical framework to guide interventions, resulting in a lack of long-term motivation for patients to maintain healthy behaviors.

Health economic evaluation illustrated that physician-pharmacist collaborative models significantly reduced the total cost of diabetes care for patients while improving patient outcomes. This provided solid evidence to support the implementation of physician-pharmacist collaborative models in primary health care. Combined with care-seeking behavior, we found that the number of outpatient visits increased by 78.7% and emergency visits decreased by half in physician-pharmacist collaborative models, indicating that the direct costs due to hospitalization for diabetes were greatly reduced [44]. In conclusion, it is economically feasible to implement physician-pharmacist collaborative clinics for diabetes management in primary health care settings.

In contrast to the previous study, significant reductions in WC and WHR, rather than BMI, were identified over 12 months in physician-pharmacist collaborative care models [45]. This inconsistency could be attributed to the peculiar physical conditions of the Chinese population, which leads to excessive fat deposition in the abdominal region instead of the peripheral region [46]. In China, the obese population primarily exhibits central obesity despite having a normal BMI, and waist-related indicators more accurately reflect obesity trends in this population [47,48]. In our study, the mean BMI at baseline was close to the normal range (BMI<24), and excessive WC was more common (WC≥90 cm for men and WC≥85 cm for women). This study suggests that physician-pharmacist collaborative models achieved significant improvements in WC and WHR, indicating that patients with diabetes and central obesity may benefit more from collaborative clinics.

In addition, our findings supported that physician-pharmacist collaborative models improve blood pressure, TC, and triglyceride levels and significantly reduce the 10-year ASCVD risk. However, we failed to find a significant enhancement in LDL-C, which is inconsistent with previous studies [49]. This discrepancy may be attributed to the interaction of our intervention focus and population characteristics. First, the primary focus of our multifaceted intervention was comprehensive glycemic control; lipid management was addressed through education but was not part of a protocol-driven intervention. Second, consistent with this context, the enrolled population in these primary care settings had a relatively favorable lipid profile, with mean LDL-C already near the recommended target for low-risk patients (LDL-C [without ASCVD]<2.6 mmol/L) [24,50,51]. Studies suggested that pharmacist interventions have more modest effects on LDL-C in such populations [52]. Overall, the collaborative models led to integrated improvements and facilitated effective, comprehensive health management.

### Strengths, Generalizability, and Implementation Considerations

This study has several strengths. First, as one of the few implementation studies on physician-pharmacist collaborative clinics with a long-term follow-up, we developed and evaluated a collaborative model for underresourced settings. A key innovation was the integration of the mHealth component, which enabled continuous support and education, overcoming geographical barriers and facilitating sustained patient engagement beyond traditional, infrequent clinic visits. While this model proved effective in primary care settings in China, its broader application requires consideration of local contexts. Key factors may include the existing health care system, cultural acceptance of team-based care, and digital tools. Second, the physician-pharmacist collaborative clinics potentially bridge the rural-urban gap in medical resources disparities for diabetes management. Moreover, the physician-pharmacist collaborative care model could serve as an evidence-based management model for patients with chronic diseases and a collaborative model for team-based care in underresourced settings. In future implementations, a pilot phase should be given priority so that the core components, structured collaboration and digitally augmented support, can be adapted to local resources and patient needs.

### Limitations

This study had several limitations. First, although the rate was comparable between the intervention and control groups, and baseline characteristics were similar between intention-to-treat and per-protocol populations, attrition bias cannot be entirely ruled out. Second, unobserved confounding bias could not be excluded from this study. Potential sources include differences in medication adherence or undisclosed lifestyle changes, as well as unmeasured variations in local health care resources or practice patterns. Third, this study was affected by the COVID-19 pandemic, which reduced patients’ willingness to seek care and introduced unpredictable bias. Fourth, the sample size was calculated on the strength of the primary outcome; therefore, we may not have sufficient power to detect other meaningful, significant outcomes. Blinding was not feasible in this study, and a Hawthorne effect may have occurred. Finally, while the final 6 study centers were randomly selected and allocated, the initial identification of candidate centers relied on a pragmatic criterion, which may introduce selection bias.

In conclusion, this trial provides strong evidence to support the implementation of the physician-pharmacist collaborative care model in long-term diabetes treatment and management in primary health care settings in underresourced areas. Patients with T2D, especially those with central obesity or high 10-year ASCVD risk, may benefit more from physician-pharmacist collaborative clinics and should be the focus of further widespread implementation. This theory-based intervention program is a promising multidisciplinary model for improving the quality and efficiency of diabetes treatment and management, as well as other chronic diseases in underresourced settings globally.

## Data Availability

The datasets generated or analyzed during this study are available from the corresponding author on reasonable request.

## Appendix

Multimedia Appendix 1 CONSORT checklist.

Multimedia Appendix 2 Fidelity checklist.

Multimedia Appendix 3 Methods and results.

## Abbreviations

ASCVD: atherosclerotic cardiovascular disease
CONSORT: Consolidated Standards of Reporting Trials
FBG: fasting blood glucose
GEE: generalized estimating equation
HbA_1c_: glycosylated hemoglobin
LDL-C: low-density lipoprotein cholesterol
mHealth: mobile health
PBG2h: 2-hour postprandial blood glucose
T2D: type 2 diabetes
TC: total cholesterol
TPB: theory of planned behavior
WC: waist circumference
WHR: waist-to-hip ratio
