# Supplementary material for: Effect of a Digital-Driven Physician-Pharmacist Collaborative Model for Diabetes in Primary Health Care: Cluster Randomized Trial
Source: J Med Internet Res. 2026 Mar 13;28:e77470. doi: 10.2196/77470 (PMC13032096; doi:10.2196/77470)
Supplement: Multimedia Appendix 1 [file jmir_v28i1e77470_app1.pdf]

Checklist of information to include when reporting a cluster randomised crossover trial

**Section** / Topic	Item no.	Checklist item	Page no.	Section heading*
**Title and Abstract**				
	1a	Identification as a cluster randomised crossover trial in the title.	1	Title
	1b	Structured summary of the trial design, methods, results, and conclusions (see separate CRXO checklist for abstracts)	2	Abstract
**Introduction**				
Background and objectives	2a	Scientific background and explanation of rationale.	3-4	Introduction
	2b	Specific objectives or hypotheses.	3-4	Introduction
**Methods**				
Trial design	3a.1	Rationale for a cluster crossover design.	4-5	Study design
	3a.2	Description of the realised trial design including:<br>• number of treatment conditions;<br>• definition of cluster (i.e. the unit of randomisation);<br>• number and duration of periods;<br>• number and composition of sequences (e.g. ABAB, BABA);<br>• number of clusters randomised to each sequence;<br>• duration of any washout periods;<br>• whether the participants assessed in different periods are the same people, different people, or a mixture of the two; and<br>• consideration of potential for carryover effects.<br>A diagram of the trial is recommended when there are more than two periods and/or treatment conditions.	4-7	Study design, Randomization and masking, Intervention and procedures
	3b	Important changes to planned methods after trial commencement (such as eligibility criteria), with reasons.	N	
Participants	4a	Eligibility criteria for clusters and participants.	5	Participants
	4b	Settings and locations where the data were collected.	5	Participants & Table 1
Intervention	5	The treatment conditions with sufficient details to allow replication, and whether they were delivered at the level of the cluster, the individual, or both.	5-6	Intervention and procedures

**Section** / Topic	Item no.	Checklist item	Page no.	Section heading*
Outcomes	6a	Completely defined pre-specified primary and secondary outcome measures, including how and when they were assessed (for specific guidance see CONSORT for outcomes).	6-7	Outcomes
	6b	Any changes to trial outcomes after the trial commenced, with reasons.	6-7	Outcomes
Sample size	7a	How sample size was determined. Method of calculation and relevant parameters with sufficient detail so the calculation can be reproduced. Assumptions made about correlations between outcomes of participants from the same cluster (see separate CRXO checklist for sample size items).	7	Sample size
	7b	When applicable, explanation of any interim analyses and stopping guidelines.	N	
Randomisation:				
Schedule generation	8a	Method used to generate the random allocation schedule.	5	Randomization and masking
	8b	Type of randomisation; details of any restricted randomisation, if used.	5	Randomization and masking
Allocation concealment mechanism	9	Specification that allocation was based on clusters; description of any methods used to conceal the allocation from the clusters until after their recruitment.	5	Randomization and masking
Implementation	10a	Who generated the random allocation schedule, who enrolled clusters, and who assigned clusters to sequences of treatments in the schedule.	5	Randomization and masking
	10b	Mechanism by which individual participants were included in clusters for the purposes of the trial (such as complete enumeration or random sampling; continuous recruitment or ascertainment, or recruitment at a fixed point in time), including who recruited or identified participants.	5	Randomization and masking
	10c	Whether consent was sought, from whom, when and for what; whether this differed between treatment conditions. Justification for any waiver or modification of informed consent requirements.	5	Randomization and masking
Blinding	11a	Who was blinded after assignment to sequences (e.g. cluster level participants, individual level participants, those assessing outcomes) and how.	5	Randomization and masking
	11b	If relevant, description of the similarity of interventions.	5	Randomization and masking

**Section** / Topic	Item no.	Checklist item	Page no.	Section heading*
Statistical methods	12a	Target estimand for each primary and secondary outcome including whether it pertains to the cluster-level or individual-level; statistical methods for their estimation including how period effects, clustering and repeated measures were taken into account. Any assessment of carryover effects should be reported.	7-8	Statistical analysis
	12b	Methods for additional analyses, such as subgroup analyses, sensitivity analyses, and adjusted analyses.	7-8	Statistical analysis
**Results**				
Participant flow (a diagram is strongly recommended)	13a	The numbers of clusters that were assessed for eligibility and were randomly assigned to each sequence. For each sequence-period (i.e. each cell) or treatment condition: • the numbers of clusters that received intended treatments and were analysed for the primary outcome; and, • the numbers of participants who were assessed for eligibility, received intended treatments and were analysed for the primary outcome.	8	Baseline characteristics & Table 1
	13b	For each sequence-period (i.e. each cell) or treatment condition, losses and exclusions for both clusters and participants with reasons.	9-10	Sensitivity analysis
Recruitment	14a	Dates of treatment periods and washout periods.	8	Baseline characteristics & Table 1
	14b	Why the trial ended or was stopped.	8	Baseline characteristics & Table 1
Baseline data	15	A table showing baseline cluster level characteristics by sequence, and individual level characteristics for each sequence-period (i.e. each cell) or treatment condition.	8	Baseline characteristics
Numbers analysed	16	The number of observations and clusters included in each analysis for each treatment condition and whether the analysis was according to the allocated schedule.	8	Baseline characteristics & Table 1
Outcomes and estimation	17a	For each primary and secondary outcome, summary statistics by sequence-period (i.e. each cell) or treatment condition; the estimated effect size and its precision (e.g. 95% confidence interval); and any within-cluster correlations or variance components estimated in the analysis.	8-9	Primary outcomes, Secondary outcomes & economic evaluation

**Section** / Topic	Item no.	Checklist item	Page no.	Section heading*
	17b	For binary outcomes, presentation of both absolute and relative effect sizes is recommended.	N	
Ancillary analyses	18	Results of any other analyses performed, including subgroup analyses, sensitivity analyses, and adjusted analyses, distinguishing pre-specified from exploratory.	10	Sensitivity analysis
Harms	19	Important harms or unintended effects in each treatment condition (for specific guidance see CONSORT Harms 2022 statement).	10	Sensitivity analysis
**Discussion**				
Limitations	20	Trial limitations, addressing sources of potential bias, imprecision, and if relevant, multiplicity of analyses. Consider potential carry-over effects.	13	Discussion
Generalisability	21	Generalisability (external validity, applicability) of the trial findings. Generalisability to clusters or individual participants, or both (as relevant).	13	Discussion
Interpretation	22	Interpretation consistent with results, balancing benefits and harms, and considering other relevant evidence.	10-13	Discussion
**Other information**				
Registration	23	Registration number and name of trial registry, or state the trial was not registered.	2	Abstract
Protocol	24	Where the full trial protocol and statistical analysis plan can be accessed, if available.	4-5	Study design
Funding	25	Sources of funding and other support (such as supply of drugs), role of funders.	15	Funding/Support
Research ethics review	26	Whether the study was approved by a research ethics committee, with identification of the review committee(s).	8	Ethical considerations
Data sharing	27	Where the individual de-identified participant data (including data dictionary), statistical code, and any other relevant documents/materials can be accessed.	14	Data availability statement
Patient and public involvement	28	Details of any patient and/or public involvement in the design, conduct and reporting of the trial; and, when applicable, other stakeholders' involvement.	14	Patient and public involvement

Cite as: McKenzie JE, Taljaard M, Hemming K, et al. Reporting of cluster randomised crossover trials: extension of the CONSORT 2010 statement with explanation and elaboration. *BMJ* 2025;388:e080472. doi:10.1136/bmj-2024-080472

* Completing section headings may be preferable to page numbers in some circumstances given headings are more likely to be persistent throughout the peer review process.
