# Supplementary material for: Effect of a Digital-Driven Physician-Pharmacist Collaborative Model for Diabetes in Primary Health Care: Cluster Randomized Trial
Source: J Med Internet Res. 2026 Mar 13;28:e77470. doi: 10.2196/77470 (PMC13032096; doi:10.2196/77470)
Supplement: Multimedia Appendix 2 [file jmir_v28i1e77470_app2.pdf]

Multimedia Appendix 2 Fidelity Checklist

Observer-Report

Kindly Note: The checklist is adapted from the general fidelity monitoring checklist for observers, developed by the Prevention Research Center, Colorado State University[1].

Background

Implementation fidelity is the extent to which an intervention is delivered as intended, which is a part of program integrity. Stated differently, intervention fidelity is the match between the intervention as it was intended to be delivered and the intervention as it is actually delivered in real-world circumstances [2]. Physician-pharmacist collaborative clinics is a well-recognized model in tertiary medical institutions in China, where pharmacists act as assistants to physicians in determining therapeutic plans and provide medication counseling and disease education to patients. Our multicenter randomized controlled study aims to establish and evaluate physician-pharmacist collaborative clinics for diabetes management in primary healthcare centers in China. The core intervention in physician-pharmacist collaborative clinics combines face-to-face follow-up visits every three months and mHealth intervention every week. As a multifaceted, multicenter intervention, we built in implementation evaluation to detect the association between interventions as designed and the intended outcomes. As physician-pharmacist collaborative care model was implemented, this implementation fidelity checklist was detected by well-trained observers monthly.

Criteria[3]

Describe the core components of the intervention and basis in theory.
To be ready for scaling up for delivery of the program, training for implementing core components must be available.
Report on local adaptations of the intervention.
Factors related to the quality of implementation should be measured and reported.

Checklist

Program Name:	
Facilitator Name(s):	
Observer Name(s):	
Date of Session:	
Session Number:<br><br>Session Title:<br><br>Session Start Time:<br><br>Session End Time:	Session Location:<br><br>Total Number of Participants:<br><br>Number of participants arriving late:<br><br>Number of participants leaving early:

**Section 1: Adherence**<br>The extent to which core components of a program are utilized (i.e., using original instructional techniques and procedures)					
1	During this session, program materials (including videos, readings, posters, slideshows, etc.) were delivered as specified in the program manual and session plan.	1-Strongly Disagree	2-Disagree	3-Agree	4-Strongly Agree
2	During this session, how often were any program activities (including worksheets, handouts, videos, games, etc.) omitted or removed?	1-Most of the time	2-Some of the time	3-Seldom	4-Never
3	During this session, were any program activities (such as worksheets, handouts,	1-Most of the time	2-Some of the time	3- Seldom	4-Never

	videos, games, etc.) replaced with an alternative activity?				
4	During this session, were any activities or resources added to the program as supplemental/additional materials?	1-Most of the time	2-Some of the time	3- Seldom	4-Never
**Notes:**					
**Total Points =**		**Maximum Points = 16**			
**Average (%) =**		To get the average percentage, divide your total points by the maximum points			

**Section 2: Exposure**<br>i.e., dosage—number, frequency, and length of program sessions					
1	Participant attendance was logged for this session.	1-Strongly Disagree	2-Disagree	3-Agree	4-Strongly Agree
2	The activities in this session were delivered in the sequence intended by the program developers.	1-Strongly Disagree	2-Disagree	3-Agree	4-Strongly Agree
3	The delivery of this session took the amount of time as intended by the program developers. (Note that this is not the same as the time intended by your organization.) *→Provide notes in the section below about how the surplus or shortage of time was compensated.*	1-Strongly Disagree	2-Disagree	3-Agree	4-Strongly Agree
**Notes:**					
**Total Points =**		**Maximum Points = 12**			
**Average (%) =**		To get the average percentage, divide your total points by the maximum points			

**Section 3: Quality of Delivery**<br>The quality and thoroughness in preparation, skills, leadership, and enthusiasm					
1	During this session, all supplies/ materials that are indicated in the program manual were available.	1-Strongly Disagree	2-Disagree	3-Agree	4-Strongly Agree
2	During this session, the amount of physical space was adequate for this session (For virtual settings refer to internet accessibility).	1-Strongly Disagree	2-Disagree	3-Agree	4-Strongly Agree
3	During this session, the quality of space was adequate for this session (For virtual settings refer to the quality of virtual space).	1-Strongly Disagree	2-Disagree	3-Agree	4-Strongly Agree
4	During this session, the facilitator(s) appeared to be prepared with the skills/knowledge to facilitate this Session.	1-Strongly Disagree	2-Disagree	3-Agree	4-Strongly Agree
5	During this session, the facilitator(s) demonstrated enthusiasm and topic interest during this session.	1-Strongly Disagree	2-Disagree	3-Agree	4-Strongly Agree

6	During this session, the facilitator(s) demonstrated positive leadership and a positive attitude.	1-Strongly Disagree	2-Disagree	3-Agree	4-Strongly Agree
7	During this session, the facilitator(s) provided a welcoming greeting when the participants entered the room.	1-Strongly Disagree	2-Disagree	3-Agree	4-Strongly Agree
8	During this session, the facilitator(s) clearly explained topics and activities.	1-Strongly Disagree	2-Disagree	3-Agree	4-Strongly Agree
9	During this session, the facilitator(s) appeared to feel comfortable interacting with the participants.	1-Strongly Disagree	2-Disagree	3-Agree	4-Strongly Agree
10	During this session, you are confident that the facilitator(s) provided high-quality facilitation of this session.	1-Strongly Disagree	2-Disagree	3-Agree	4-Strongly Agree
**Notes:**					

**Total Points =**	**Maximum Points = 40**
**Average (%) =**	To get the average percentage, divide your total points by the maximum points

**Section 4: Participant Responsiveness**<br>The participants' engagement with the program					
1	During this session, participants were interested in the material.	1-Strongly Disagree	2-Disagree	3-Agree	4-Strongly Agree
2	During this session, participants were engaged in the material.	1-Strongly Disagree	2-Disagree	3-Agree	4-Strongly Agree
3	During this session, members were participating in the discussions/ activities.	1-Strongly Disagree	2-Disagree	3-Agree	4-Strongly Agree
4	During this session, participants understood/comprehended the material. *→Elaborate & provide examples in the notes below.*	1-Strongly Disagree	2-Disagree	3-Agree	4-Strongly Agree
**Notes:**					

**Total Points =**	**Maximum Points = 16**
**Average (%) =**	To get the average percentage, divide your total points by the maximum points

Final Notes on Special Circumstances:

In addition to the notes above, please describe any unusual circumstances that arose (such as disturbances) that might have affected the group's dynamics for this session:

Total Points Recap:
Section 1: Adherence → Average (%)=
Section 2: Exposure → Average (%)=
Section 3: Quality of Delivery → Average (%)=
Section 4: Participant Responsiveness → Average (%)=
Overall Fidelity* =
*** Add the total points of all 4 sections together and divide by the maximum points.**

Interpretation:
The Implementation Fidelity Monitoring

Fidelity	Statues
25%-59%	Implementation Failure
60%-74%	Implementation Needs Improvement
75%-100%	Adequate Implementation

[1] Prevention Research Center, Colorado State University. Fidelity Monitoring Checklist (Observer-Report) URL: https://www.chhs.colostate.edu/prc/implementation-toolbox/resources/fidelity-monitoring-checklist-observer-report/ [accessed 2023-08-30]
[2] Hill LG, Maucione K, Hood BK. A focused approach to assessing program fidelity. Prev Sci. 2007 Mar;8(1):25-34. doi: 10.1007/s11121-006-0051-4.
[3] Gottfredson DC, Cook TD, Gardner FE, Gorman-Smith D, Howe GW, Sandler IN, Zafft KM. Standards of Evidence for Efficacy, Effectiveness, and Scale-up Research in Prevention Science: Next Generation. Prev Sci. 2015 Oct;16(7):893-926. doi: 10.1007/s11121-015-0555-x.
