# Supplementary material for: Effect of a Digital-Driven Physician-Pharmacist Collaborative Model for Diabetes in Primary Health Care: Cluster Randomized Trial
Source: J Med Internet Res. 2026 Mar 13;28:e77470. doi: 10.2196/77470 (PMC13032096; doi:10.2196/77470)
Supplement: Multimedia Appendix 3 [file jmir_v28i1e77470_app3.pdf]

Supplementary material 3 Methods and results

Table S1. Training courses for pharmacists in the intervention group

Table S2. Services to be provided by pharmacists in the intervention group

Table S3. Comprehensive control target of type 2 diabetes in China

Table S4. Characteristics of enrolled county hospitals

Table S5. The rates of control on clinical outcomes at baseline and 12-month

Table S6. The 12-month effectiveness of the physician-pharmacist collaborative clinics on clinical outcomes in modified ITT population

Table S7. The difference of demographic characteristics between ITT population and PP population

Table S8. Demographic characteristics in PP population

Table S9. The 12-month effectiveness of the physician-pharmacist collaborative clinics on clinical outcomes in PP population

Table S10. The 12-month total costs of patients in the two groups in PP population

Table S1. Training courses for pharmacists in the intervention group

Training courses	Details	time
**Diabetes management**	1.Theoretical knowledge<br>☐ Basic knowledge of diabetes, including identification of diabetes complications<br>☐ Basic knowledge of hypoglycemic agents（oral & injection medicine）<br>☐ treatment target of blood glucose level<br>☐ Laboratory tests interpretation<br>☐ Medication adherence<br>☐ Diet<br>☐ Exercise<br>☐ lifestyle<br>2. Practice and Application in patients<br>3. Six tests to assess their theoretical knowledge and practice skills	Two months
**Cooperative skills**	1. Theoretical knowledge on communication skills<br>2. Practice in outpatient clinics and inpatient wards	One month

Table S2. Services to be provided by pharmacists in the intervention group

Services	Details	Methods
**Diabetes education**	☐ Disease introduction<br>☐ Control target of blood glucose<br>☐ The types and principles of hypoglycemic agents<br>☐ Self-monitoring of blood glucose	☐ Educate patients of diabetes management in the collaborative clinic. The knowledge will be printed out and given to patients for home study.<br>☐ Create a WeChat group with all the enrolled patients in, and pharmacists will share diabetes knowledge periodically.
**Medication guidance**	☐ The importance of medication adherence<br>☐ Administration methods（oral & injection medication）<br>☐ Administration timing<br>☐ The injection technique of insulin<br>☐ Drug-drug interaction<br>☐ Drug storage	
**Lifestyle intervention**	☐ Exercise<br>☐ Diet	☐ Educate patients in the collaborative clinic.<br>☐ Help patients make a healthy lifestyle plan.
**ADR * & complications identification and treatment**	☐ Recognition and prevention of hypoglycemia<br>☐ Identification and processing of complications of diabetes<br>☐ Identification and processing of ADR	☐ Educate patients in the collaborative clinic.<br>☐ Call the pharmacist if an emergency event occurs

*ADR: adverse drug reaction

Table S3. Comprehensive control target of type 2 diabetes in China

Variables	Target of Control [1]
BMI (Kg/m^2)	<24.0
Waist circumference (cm)	
Male	<90
Female	<85
Waist-hip ratio	
Male	<0.9
Female	<0.85
Systolic blood pressure (mmHg)	<130
Diastolic blood pressure (mmHg)	<80
Glycosylated hemoglobin (%)	<7.0
Blood glucose (mmol/L)	
Fasting blood glucose	4.4-7.0
Postprandial blood glucose	≤10.0
Triglyceride (mmol/L)	<1.7
Total cholesterol (mmol/L)	<4.5
High-density lipoprotein (mmol/L)	
Male	>1.0
Female	>1.3
Low-density lipoprotein (mmol/L)	<2.6

[1] Branch. CMAD. Guidelines for the Prevention and Treatment of Type 2 Diabetes in China (2020 Edition). Chinese Journal of Practical Internal Medicine. 2021;41:757.

Table S4. Characteristics of enrolled county hospitals

Variable	Center 1	Center 2	Center 3	Center 4	Center 5	Center 6
Location	Central	East	North	West	North	East
GDP Per capita	High	High	Medium	Medium	Low	Low
Major ethnic groups	Han	Han	Han	Han	Han	Han
Total population (*10,000)	126.56	86.12	85.56	75.50	86.09	98.28
Monthly diabetic outpatient numbers	600	600	300	280	260	130

Table S5. The rates of control on clinical outcomes at baseline and 12-month

Variables	Intervention		Control		*P*
	n	Rate of control [a]	n	Rate of control	
**BMI**					.494
Baseline	123	42.27%	99	34.98%	
12 months	143	49.14%	108	38.16%	
**WC**					<.001
Baseline	132	45.36%	107	37.81%	
12 months	169	58.08%	81	28.62%	
Male					
Baseline	67	48.20%	66	40.74%	
12 months	86	61.87%	51	31.48%	
Female					
Baseline	65	42.76%	41	33.88%	
12 months	83	54.61%	30	24.79%	
**WHR**					<.001
Baseline	69	23.71%	59	20.85%	
12 months	104	35.74%	39	13.78%	
Male					
Baseline	48	16.49%	42	14.84%	
12 months	68	23.37%	29	10.25%	
Female					
Baseline	21	7.22%	17	6.01%	
12 months	36	12.37%	10	3.53%	
**SBP**					.007
Baseline	161	55.33%	141	49.82%	
12 months	204	70.10%	148	52.30%	
**DBP**					<.001
Baseline	190	65.29%	168	59.36%	
12 months	225	77.32%	176	62.19%	
**HbA_{1c}**					.010
Baseline	0	0.00%	5	1.77%	
12 months	156	53.61%	109	38.52%	
**FBG**					.042
Baseline	45	15.46%	56	19.79%	
12 months	135	46.39%	107	37.81%	
**PBG2h**					<.001
Baseline	6	2.06%	10	3.53%	
12 months	103	35.40%	44	15.55%	
**TG**					.036
Baseline	111	38.14%	110	38.87%	
12 months	158	54.30%	115	40.64%	
**TC**					.045
Baseline	98	33.68%	116	40.99%	
12 months	153	52.58%	119	42.05%	
**HDL**					.019
Baseline	161	55.33%	156	55.12%	
12 months	201	69.07%	136	48.06%	
Male					
Baseline	97	69.78%	109	67.28%	
12 months	118	84.89%	92	56.79%	
Female					
Baseline	64	42.11%	47	38.84%	
12 months	83	54.61%	44	36.36%	
**LDL**					.194
Baseline	97	33.33%	112	39.58%	
12 months	110	37.80%	114	40.28%	

[a] Standard rate of control was referred to the comprehensive control target of type 2 diabetes in China (2020 Edition) (Supplement S2); *HbA_{1c} (%)*, glycosylated hemoglobin; *ASCVD*, atherosclerotic cardiovascular disease; *FBG (mmol/l)*, fasting blood glucose; *PBG2h (mmol/l)*, 2-hour postprandial blood glucose; *BMI (kg/m2)*, body mass index; *WC (cm)*, waist circumference; *WHR*, waist-hip ratio; *SBP (mmHg)*, systolic blood pressure; *DBP (mmHg)*, diastolic blood pressure; *TG (mmol/l)*, triglyceride; *TC (mmol/l)*, total cholesterol; *HDL-C (mmol/l)*, high-density lipoprotein; *LDL-C (mmol/l)*, low-density lipoprotein.

Table S6. The 12-month effectiveness of the physician-pharmacist collaborative clinics on clinical outcomes in modified ITT population

Variables	Intervention group	Control group	Unadjusted Effect			Adjusted Effect [b]		
			Time	Group	Group*Time [a]	Time	Group	Group*Time
**BMI**			<.001	.062	.218	<.001	.109	.234
Baseline	24.73±3.38	25.02±3.28						
3 months	24.49±3.22	24.68±3.38						
6 months	24.37±3.13	24.89±3.16						
9 months	24.23±3.12	24.73±3.29						
12 months	24.28±3.14	24.77±3.36						
**WC**			<.001	<.001	<.001	<.001	0.339	<.001
Baseline	89.83±9.68	90.98±9.89						
3 months	89.06±9.67	91.46±9.83						
6 months	87.09±9.21	91.82±9.46						
9 months	87.02±9.42	92.50±9.13						
12 months	86.84±9.24	91.92±9.85						
**WHR**			<.001	<.001	<.001	<.001	378	.008
Baseline	0.926±0.069	0.929±0.070						
3 months	0.920±0.073	0.930±0.080						
6 months	0.911±0.068	0.932±0.073						
9 months	0.909±0.070	0.938±0.073						
12 months	0.910±0.068	0.933±0.067						
**SBP**			<.001	.001	.007	<.001	.378	.008
Baseline	132.53±16.05	134.26±20.41						
3 months	130.21±13.26	132.67±16.37						
6 months	128.53±12.14	132.92±14.10						
9 months	127.28±11.24	134.30±17.08						
12 months	127.30±11.70	132.54±15.20						
**DBP**			<.001	<.001	.014	<.001	.886	.018
Baseline	80.47±9.03	81.96±10.71						
3 months	78.72±9.04	81.77±10.64						
6 months	77.51±8.16	81.53±9.45						
9 months	77.12±8.99	82.09±9.94						
12 months	77.06±7.94	81.26±8.52						
**HbA1c**			<.001	<.001	<.001	<.001	.636	<.001
Baseline	9.65±1.87	10.02±2.15						
3 months	7.93±1.53	8.43±2.19						
6 months	7.76±1.63	8.15±1.86						
9 months	7.55±1.48	8.18±1.87						
12 months	7.08±1.13	8.06±2.02						
**FBG**			<.001	.015	.295	<.001	<.001	.273
Baseline	10.60±3.60	10.69±4.66						
3 months	8.38±2.54	9.03±3.32						
6 months	8.40±2.77	8.92±2.67						
9 months	8.02±2.43	8.31±2.28						
12 months	7.58±2.09	8.22±2.67						
**PBG2h**			<.001	.988	.031	<.001	.001	.039
Baseline	15.34±4.64	15.03±5.84						
3 months	12.38±3.89	12.03±4.54						
6 months	11.60±3.67	11.45±4.63						
9 months	11.36±3.42	11.06±4.07						
12 months	10.44±3.66	11.17±4.10						
**TG**			<.001	.826	.015	<.001	.209	.018
Baseline	3.09±3.87	2.53±2.20						
3 months	2.52±2.54	2.40±2.37						
6 months	2.37±2.24	2.44±2.70						
9 months	2.19±2.30	2.20±1.53						

12 months	1.93±1.40	2.24±1.53						
**TC**			<.001	.882	.001	<.001	.018	.001
Baseline	5.12±1.74	4.84±1.36						
3 months	4.71±1.18	4.60±1.09						
6 months	4.43±0.96	4.47±1.14						
9 months	4.16±0.77	4.28±1.12						
12 months	4.12±0.86	4.37±0.96						
**HDL-C**			.997	.081	.248	.999	.555	.284
Baseline	1.30±0.53	1.30±0.60						
3 months	1.33±0.66	1.26±0.37						
6 months	1.32±0.45	1.24±0.49						
9 months	1.33±0.49	1.25±0.49						
12 months	1.36±0.39	1.25±0.56						
**LDL-C**			.003	.851	.403	.004	.200	.420
Baseline	2.96±1.25	2.92±0.94						
3 months	2.74±0.96	2.85±0.93						
6 months	2.78±0.95	2.78±0.81						
9 months	2.79±0.93	2.77±0.77						
12 months	2.78±0.83	2.78±0.80						
**ASCVD risks**			<.001	.001	<.001	<.001	.372	<.001
Baseline	7.05±4.92	8.06±6.65						
12 months	5.70±4.05	8.07±6.37						
**Satisfaction**				<.001			<.001	
12 months	32.43±4.38	26.16±5.59						

[a] Estimated group by time interaction effects from generalized estimation equation.

[b] Adjusted for center, gender, age, comorbidity, history of smoking and alcohol, family history, education, income, occupation and residence; *HbA_{1c} (%)*, glycosylated hemoglobin; *ASCVD*, atherosclerotic cardiovascular disease; *FBG (mmol/l)*, fasting blood glucose; *PBG2h (mmol/l)*, 2-hour postprandial blood glucose; *BMI (kg/m2)*, body mass index; *WC (cm)*, waist circumference; *WHR*, waist-hip ratio; *SBP (mmHg)*, systolic blood pressure; *DBP (mmHg)*, diastolic blood pressure; *TG (mmol/l)*, triglyceride; *TC (mmol/l)*, total cholesterol; *HDL-C (mmol/l)*, high-density lipoprotein; *LDL-C (mmol/l)*, low-density lipoprotein.

Table S7. The difference of demographic characteristics between ITT population and PP population [a]

Characteristics	PP population (N=510)	ITT population (N=574)	*P*
**Age, years**	55.0 (48.0-63.0)	55.0 (48.0-64.0)	.95
**Gender**			.33
Male	279 (54.7%)	301 (52.4%)	
Female	231 (45.3%)	273 (47.6%)	
**Comorbidity**			
hypertension			.99
Yes	79 (15.5%)	90 (15.7%)	
hyperlipidemia			.72
Yes	15 (1.2%)	26 (4.5%)	
coronary heart disease			.34
Yes	15 (2.9%)	20 (3.5%)	
**History of smoking**			.63
Yes	174 (34.1%)	195 (34.0%)	
**History of alcohol**			.67
Yes	140 (27.5%)	156 (26.7%)	
**Family history**			
diabetes			.98
Yes	154 (30.2%)	164 (28.6%)	
hypertension			.86
Yes	90 (17.6%)	94 (16.4%)	
hyperlipidemia			.96
Yes	38 (7.5%)	45 (7.8%)	
**Education**			.87
Junior high school and below	282 (55.3%)	318 (55.4%)	
Senior high school	133 (26.1%)	152 (26.5%)	
College and above	95 (18.6%)	104 (18.1%)	
**Income per month (CNY)[a]**			.97
<1000	91 (17.8%)	96 (16.7%)	
1000-3000	166 (32.6%)	191 (33.3%)	
>3000	253 (49.6%)	287 (50.0%)	
**Occupation**			.92
Full-time job	363 (71.2%)	395 (68.8%)	
Part-time job or no job	147 (28.8%)	179 (31.2%)	
**Residence**			.87
Rural area	280 (54.9%)	321 (55.9%)	
Town or Urban area	230 (45.1%)	253 (44.1%)	
**BMI**	24.84±3.44	24.87±3.33	.77
**WC**			.94
Male	92.08±9.38	91.92±9.34	
Female	88.50±10.40	88.73±10.04	
**WHR**			.82
	0.928±0.061	0.927±0.062	
	0.924±0.077	0.927±0.078	
**SBP**	133.44±18.27	133.38±18.33	.91
**DBP**	81.14±9.93	81.20±9.90	.89
**HbA1c**	9.86±2.03	9.83±2.02	.84
**FBG**	10.58±4.14	10.65±4.16	.81
**PBG2h**	14.86±5.10	15.19±5.27	.34
**TG**	2.82±3.28	2.80±3.15	.67
**TC**	4.98±1.57	4.98±1.56	.95
**HDL-C**	1.30±0.57	1.30±0.57	.95
**LDL-C**	2.93±1.11	2.94±1.11	.88
**ASCVD risks**	7.62±6.05	7.56±5.87	.93

[a] Per-protocol (PP) population includes patients who followed the protocol and were not lost to follow-up; intention-to-treat (ITT) population includes all patients who participated in this trial, modified to exclude 26 patients who withdrew after randomization but before the trial implemented; 1 CNY≈0.14 USD; *HbA_{1c} (%)*, glycosylated hemoglobin; *ASCVD*, atherosclerotic cardiovascular disease; *FBG (mmol/l)*, fasting blood glucose; *PBG2h (mmol/l)*, 2-hour postprandial blood glucose; *BMI (kg/m2)*, body mass index; *WC (cm)*, waist circumference; *WHR*, waist-hip ratio; *SBP (mmHg)*, systolic blood pressure; *DBP (mmHg)*, diastolic blood pressure; *TG (mmol/l)*, triglyceride; *TC (mmol/l)*, total cholesterol; *HDL-C (mmol/l)*, high-density lipoprotein; *LDL-C (mmol/l)*, low-density lipoprotein.

Table S8. Demographic characteristics in PP population

Characteristics	Overall (N=510)	Intervention (n=276)	Control(n=234)
**Age, years**	55.0 (48.0-63.0)	55.5 (46.3-67.0)	54.0(49.0-60.0)
**Gender**	510		
Male	279 (54.7%)	134 (48.6%)	145 (62.0%)
Female	231 (45.3%)	142 (51.4%)	89 (38.0%)
**Comorbidity**			
hypertension			
Yes	79 (15.5%)	45 (16.3%)	34 (14.5%)
hyperlipidemia			
Yes	14 (2.7%)	5 (1.8%)	9 (3.8%)
coronary heart disease			
Yes	15 (2.9%)	8 (2.9%)	7 (3.0%)
**History of smoking**			
Yes	174 (34.1%)	84 (30.4%)	90 (38.5%)
No	336 (65.9%)	192 (69.6%)	144 (61.5%)
**History of alcohol**			
Yes	140 (27.5%)	77 (27.9%)	63 (26.9%)
No	370 (72.5%)	199 (72.1%)	171 (73.1%)
**Family history**			
diabetes			
Yes	154 (30.2%)	87 (31.5%)	67 (28.6%)
No	356 (69.8%)	189 (68.5%)	167 (71.4%)
hypertension			
Yes	90 (17.6%)	54 (19.6%)	36 (15.4%)
No	420 (82.4%)	222 (80.4%)	198 (84.6%)
hyperlipidemia			
Yes	38 (7.5%)	22 (8.0%)	16 (6.8%)
No	472 (92.5%)	254 (92.0%)	218 (93.2%)
coronary heart disease			
Yes	39 (7.6%)	26 (9.4%)	13 (5.6%)
No	471 (92.4%)	250 (90.6%)	221 (94.4%)
**Education**			
Junior high school and below	282 (55.3%)	160 (58.0%)	122 (52.1%)
Senior high school	133 (26.1%)	72 (26.1%)	61 (26.1%)
College and above	95 (18.6%)	44 (15.9%)	51 (21.8%)
**Income per month (CNY)**[a]			
<1000	91 (17.8%)	54 (19.6%)	37 (15.8%)
1000-3000	166 (32.6%)	89 (32.2%)	77 (32.9%)
>3000	253 (49.6%)	133 (48.2%)	120 (51.3%)
**Occupation**			
Full-time job	363 (71.2%)	205 (74.3%)	158 (67.5%)
Part-time job or no job	147 (28.8%)	71 (25.7%)	76 (32.5%)
**Residence**			
Rural area	280 (54.9%)	133 (48.2%)	97 (41.5%)
Town or Urban area	230 (45.1%)	143 (51.8%)	137 (58.5%)
**BMI**	24.84±3.44	24.66±3.36	25.06±3.53
**WC**			
Male	92.08±9.38	91.24±8.90	92.86±9.77
Female	88.50±10.40	88.68±10.34	88.31±10.67
**WHR**			
Male	0.928±0.061	0.924±0.076	0.932±0.061
Female	0.924±0.077	0.929±0.068	0.923±0.080
**SBP**	133.44±18.27	132.80±16.24	134.19±20.40
**DBP**	81.14±9.93	80.50±9.12	81.90±10.79
**HbA1c**	9.86±2.03	9.67±1.88	10.08±2.17
**FBG**	10.58±4.14	10.52±3.57	10.65±4.73
**PBG2h**	14.86±5.10	15.02±4.50	14.65±5.77
**TG**	2.82±3.28	3.08±3.94	2.53±2.31
**TC**	4.98±1.57	5.10±1.77	4.84±1.31
**HDL-C**	1.30±0.57	1.29±0.54	1.30±0.60
**LDL-C**	2.93±1.11	2.94±1.27	2.91±0.89
**ASCVD risks**	7.62±6.05	6.94±4.96	8.39±7.02

a 1 CNY≈0.14 USD; *HbA_{1c} (%)*, glycosylated hemoglobin; *ASCVD*, atherosclerotic cardiovascular disease; *FBG (mmol/l)*, fasting blood glucose; *PBG2h (mmol/l)*, 2-hour postprandial blood glucose; *BMI (kg/m2)*, body mass index; *WC (cm)*, waist circumference; *WHR*, waist-hip ratio; *SBP (mmHg)*, systolic blood pressure; *DBP (mmHg)*, diastolic blood pressure; *TG (mmol/l)*, triglyceride; *TC (mmol/l)*, total cholesterol; *HDL-C (mmol/l)*, high-density lipoprotein; *LDL-C (mmol/l)*, low-density lipoprotein.

Table S9. The 12-month effectiveness of the physician-pharmacist collaborative clinics on clinical outcomes in PP population

Variables	Intervention group	Control group	Unadjusted Effect			Adjusted Effect [b]		
			Time	Group	Group*Time [a]	Time	Group	Group*Time
**BMI**			<.001	.081	.43	<.001	.077	.42
Baseline	24.66±3.36	25.06±3.53						
3 months	24.49±3.21	24.59±3.43						
6 months	24.34±3.13	24.85±3.20						
9 months	24.22±3.10	24.68±3.37						
12 months	24.23±3.16	24.84±3.50						
**WC**			<.001	<.001	<.001	<.001	<.001	<.001
Baseline	89.88±9.76	91.12±10.34						
3 months	89.15±9.73	91.31±9.82						
6 months	87.08±9.31	91.76±9.67						
9 months	87.03±9.43	92.10±9.24						
12 months	86.87±9.31	91.68±10.15						
**WHR**			<.001	<.001	<.001	<.001	<.001	<.001
Baseline	0.924±0.069	0.929±0.068						
3 months	0.919±0.073	0.927±0.076						
6 months	0.910±0.068	0.931±0.070						
9 months	0.909±0.069	0.937±0.072						
12 months	0.909±0.068	0.931±0.064						
**SBP**			<.001	.002	.028	<.001	.002	.025
Baseline	132.80±16.24	134.19±20.40						
3 months	129.93±13.28	133.09±16.88						
6 months	128.36±12.28	132.68±14.43						
9 months	127.17±11.28	133.27±17.02						
12 months	127.10±11.70	132.55±15.34						
**DBP**			<.001	<.001	.013	<.001	<.001	.014
Baseline	80.50±9.12	81.90±10.79						
3 months	78.52±9.02	82.21±10.33						
6 months	77.48±8.23	81.59±9.27						
9 months	77.01±9.02	82.09±9.83						
12 months	76.96±7.73	81.19±8.17						
**HbA1c**			<.001	<.001	<.001	<.001	<.001	<.001
Baseline	9.67±1.88	10.08±2.17						
3 months	7.93±1.52	8.40±2.14						
6 months	7.79±1.65	8.07±1.79						
9 months	7.52±1.46	8.20±1.84						
12 months	7.06±1.10	8.07±2.03						
**FBG**			<.001	.003	.41	<.001	.002	.41
Baseline	10.52±3.57	10.65±4.73						
3 months	8.36±2.56	9.18±3.34						
6 months	8.42±2.82	9.06±2.62						
9 months	7.98±2.36	8.48±2.32						
12 months	7.50±2 .00	8.22±2.62						
**PBG2h**			<.001	.49	.044	<.001	.50	.046
Baseline	15.02±4.50	14.65±5.77						
3 months	12.30±3.87	12.27±4.61						
6 months	11.54±3.65	11.61±4.61						
9 months	11.23±3.36	11.39±4.22						
12 months	10.47±3.68	11.28±4.07						
**TG**			<.001	.61	.022	<.001	.62	.023
Baseline	3.08±3.94	2.53±2.31						
3 months	2.48±2.52	2.24±2.01						
6 months	2.36±2.28	2.26±2.28						
9 months	2.17±2.34	2.22±1.52						
12 months	1.90±1.40	2.18±1.44						

**TC**			<.001	.81	.002	<.001	.26	.002
Baseline	5.10±1.77	4.84±1.31						
3 months	4.70±1.17	4.54±1.07						
6 months	4.40±0.95	4.44±1.15						
9 months	4.14±0.75	4.28±1.12						
12 months	4.09±0.83	4.34±0.98						
**HDL-C**			.98	.17	.39	.96	.30	.42
Baseline	1.29±0.54	1.30±0.60						
3 months	1.33±0.67	1.28±0.37						
6 months	1.32±0.46	1.27±0.51						
9 months	1.33±0.50	1.24±0.43						
12 months	1.36±0.40	1.27±0.57						
**LDL-C**			.047	.45	.44	.052	.13	.42
Baseline	2.94±1.27	2.91±0.89						
3 months	2.74±0.97	2.88±0.96						
6 months	2.76±0.95	2.82±0.83						
9 months	2.79±0.93	2.85±0.75						
12 months	2.78±0.83	2.81±0.83						
**ASCVD risks**			<.001	<.001	.005	<.001	<.001	.003
Baseline	6.94±4.96	8.39±7.02						
12 months	5.64±4.11	8.15±6.55						

[a] Estimated group by time interaction effects from generalized estimation equation.

[b] Adjusted for gender, age, comorbidity, history of smoking and alcohol, family history, education, income, occupation and residence; *HbA1c (%)*, glycosylated hemoglobin; *ASCVD*, atherosclerotic cardiovascular disease; *FBG (mmol/l)*, fasting blood glucose; *PBG2h (mmol/l)*, 2-hour postprandial blood glucose; *BMI (kg/m2)*, body mass index; *WC (cm)*, waist circumference; *WHR*, waist-hip ratio; *SBP (mmHg)*, systolic blood pressure; *DBP (mmHg)*, diastolic blood pressure; *TG (mmol/l)*, triglyceride; *TC (mmol/l)*, total cholesterol; *HDL-C (mmol/l)*, high-density lipoprotein; *LDL-C (mmol/l)*, low-density lipoprotein.

Table S10. The 12-month total costs of patients in the two groups in PP population

Variables	Overall (N=510)	Intervention (n=276)	Control (n=234)	*P*
Drug cost	4,049.30±2,228.32	3,918.94±2,003.63	4,203.05±2,462.75	.11
Registration cost	47.98±29.20	57.18±28.96	37.14±25.57	<.001
Examination cost	534.97±777.38	555.23±322.39	511.50±1,088.91	.34
Hospitalization cost	1,810.53±8,111.01	653.79±2,297.19	3,174.89±11,577.36	<.001
Lost wages	173.04±283.49	176.67±306.54	168.76±254.21	.64
Transportation cost	43.73±65.34	57.25±70.38	27.78±54.84	<.001
Total cost	6,653.75±8,523.12	5,407.98±3,162.46	8,123.12±11,953.35	<.001
